# A cost-effectiveness analysis of community water fluoridation for schoolchildren

**DOI:** 10.1186/s12903-021-01490-7

**Published:** 2021-03-25

**Authors:** Jodi Cronin, Stephen Moore, Máiréad Harding, Helen Whelton, Noel Woods

**Affiliations:** 1 Centre for Policy Studies, Cork University Business School, University College Cork, Cork, Ireland; 2 Oral Health Services Research Centre, University College Cork, Cork, Ireland; 3 College of Medicine and Health, University College Cork, Cork, Ireland

**Keywords:** Cost-effectiveness analysis, Economic evaluation, Community water fluoridation, Preventive dentistry, Public health dentistry

## Abstract

**Background:**

Community water fluoridation (CWF), the controlled addition of fluoride to the water supply for the prevention of dental caries (tooth decay), is considered a safe and effective public health intervention. The Republic of Ireland (*Ireland*) is the only country in Europe with a legislative mandate for the fluoridation of the public water supply, a key component of its oral health policy. However, more recently, there has been an increase in public concern around the relevance of the intervention given the current environment of multiple fluoride sources and a reported increase in the prevalence of enamel fluorosis. The aim of this economic analysis is to provide evidence to inform policy decisions on whether the continued public investment in community water fluoridation remains justified under these altered circumstances.

**Methods:**

Following traditional methods of economic evaluation and using epidemiological data from a representative sample of 5-, 8-, and 12-year-old schoolchildren, this cost-effectiveness analysis, conducted from the health-payer perspective, compared the incremental costs and consequences associated with the CWF intervention to no intervention for schoolchildren living in Ireland in 2017. A probabilistic model was developed to simulate the potential lifetime treatment savings associated with the schoolchildren’s exposure to the intervention for one year.

**Results:**

In 2017, approximately 71% of people living in Ireland had access to a publicly provided fluoridated water supply at an average per capita cost to the state of €2.15. The total cost of CWF provision to 5-, 8-, and 12-year-old schoolchildren (n = 148,910) was estimated at €320,664, and the incremental cost per decayed, missing, or filled tooth (d_3vc_mft/D_3vc_MFT) prevented was calculated at €14.09. The potential annual lifetime treatment savings associated with caries prevented for this cohort was estimated at €2.95 million. When the potential treatment savings were included in the analysis, the incremental cost per d_3vc_mft/D_3vc_MFT prevented was -€115.67, representing a cost-saving to the health-payer and a positive return on investment. The results of the analysis were robust to both deterministic and probability sensitivity analyses.

**Conclusion:**

Despite current access to numerous fluoride sources and a reported increase in the prevalence of enamel fluorosis, CWF remains a cost-effective public health intervention for Irish schoolchildren.

**Supplementary Information:**

The online version contains supplementary material available at 10.1186/s12903-021-01490-7.

## Background

Oral diseases, despite being largely preventable, remain the most common noncommunicable diseases worldwide [1]. The Global Burden of Disease Study 2017 estimated that, of the 3.47 billion people affected by an oral disease, 2.3 billion people had caries experience in permanent teeth and 532 million children had caries in the deciduous dentition [2]. The incidence of dental caries is highly correlated to lifestyle factors and also reflects whether preventive measures such as exposure to fluoride sources and good oral hygiene are present [3]. The disease and its sequelae can cause significant pain, loss of tooth structure, diminished quality of life and are expensive to treat [4]. A recent study reported the annual economic impact of dental disease in Western Europe, was €120.46 billion in 2015,^1^ of which 64% was directly attributable to treatment costs, with the remaining 36% relating to productivity losses due to absenteeism from school and work [6]. Thus, the human, psychological and financial implications of this largely preventable disease are substantial [4].

Community water fluoridation (CWF), the controlled addition of fluoride to the public water supply (PWS), is an approved public health intervention to reduce the prevalence and severity of dental caries [7–9]. CWF is a universal intervention that delivers acceptable levels of fluoride exposure for all and can confer a positive impact across all socioeconomic groups [4]. In the US, it was recognised as one of ten great public health promotion measures of the twentieth century by virtue of its distributional cost-effectiveness at reducing oral disease [10].

The Republic of Ireland (*Ireland*) is the only country in Europe with a legislative mandate for the fluoridation of the PWS [4], a key component of its oral health policy [11]. According to the latest data from the Environmental Protection Agency Ireland (EPA), 71% of the population were in receipt of CWF from a PWS in 2017 [12]. Since its introduction to Ireland in the 1960′s, the clinical effectiveness of CWF has been closely monitored and validated [13–17]. However, more recent surveys, conducted in an environment of multiple fluoride sources, show a persistent benefit from water fluoridation, but have identified a reduction in the difference in disease levels between those with and without CWF and an increased prevalence of enamel fluorosis in both communities [16, 17]. The caries levels reported in the Irish children’s oral health survey in 2002 were lower amongst children with lifetime exposure to CWF than those without lifetime exposure to CWF (d_3vc_mft 5 years: 1.3 with CWF, 2.2 without (w/o) CWF; D_3vc_MFT 8 years: 0.4 with CWF, 0.5 w/o CWF; D_3vc_MFT 12 years: 1.4 with CWF, 1.8 w/o CWF [17]). Conversely, the prevalence of fluorosis, as measured using Dean’s Index of Fluorosis [18] was higher amongst children with CWF (the percentage of children with visible forms of fluorosis was 12% and 3% for those aged 8, with and without lifetime exposure to CWF respectively, while the corresponding figures for 12-year-olds were 16% and 6% [17]). Subsequently, the Forum on Fluoridation, established by the Department of Health to independently review CWF, issued several policy recommendations to attain maximum protection against dental caries and minimise the incidence of enamel fluorosis [19]. One recommendation, redefining the optimal level of fluoride in drinking water from 0.8–1.0 mg/L to 0.6–0.8 mg/L with a target value of 0.7 mg/L, was implemented in 2007. Another consequence of the review was the formation of the Irish Expert Body on Fluorides and Health, who commissioned an independent audit of the CWF process in Ireland [20].

It is within this context that we evaluate the economic evidence to inform policy decisions around the continuation of CWF as a national oral health policy. The argument for CWF depends on the benefits, in terms of a lower incidence of dental caries, and the potential savings generated by the reduction in need for treatment that could be returned by using the same resources in an alternative oral healthcare programme [21]. A review of the more recent economic literature identified evaluations conducted in the US [22–24], Canada [25], Australia [26–28] and New Zealand [29, 30] but did not retrieve any formal economic assessment of the Irish CWF programme. It is therefore appropriate that an evaluation is performed to understand whether the continued investment of finite public resources is justified under these altered circumstances. This research was conducted as a component of the Fluoridation and Caring for Children’s Teeth (FACCT) study [31]. The primary aim of FACCT was a valuation of health outcomes in schoolchildren following the reduction in the level of fluoride in the PWS [31].

## Methods

This cost-effectiveness analysis, guided by methodologies applied in previous analyses [21–24, 30, 32], and using epidemiological data from a representative sample of 5-, 8-, and 12-year-old schoolchildren collected in FACCT [31], compared the incremental costs and consequences associated with exposure to the CWF intervention compared to no CWF exposure for this cohort living in Ireland in 2017. The incremental cost-effectiveness ratio (ICER) of CWF was re-calculated to include the potential savings associated with the reduced need for dental treatment. A simple probabilistic model, developed in MS Excel [33], simulated the lifetime treatment savings associated with exposure to CWF during 2017. The analysis adopted the health-payer perspective (direct and indirect treatment savings) and applied the Irish recommended social discount rate of 4% [34, 35] to the potential lifetime treatment savings. The study adhered to the Consolidated Health Economic Evaluation Reporting Standards [36] for reporting economic evaluations and reports the annual CWF cost per d_3vc_mft/D_3vc_MFT prevented along with the net CWF cost (cost of CWF provision minus the expected treatment savings) per d_3vc_mft/D_3vc_MFT prevented in 2017 Euro.

### The cost of CWF provision per decayed, missing or filled tooth prevented

The cost-effectiveness of CWF was calculated as the annual ratio of incremental costs to incremental consequences in terms of caries prevented, as measured by the d_3vc_mft/D_3vc_MFT index (Eq. 1), for schoolchildren with exposure to the intervention compared to those without. The d_3vc_mft/D_3vc_MFT index records caries at the dentinal level of involvement according to WHO criteria [37], and this was expanded in FACCT [31] to include visible non-cavitated dentinal caries thus providing more detail on disease levels and the stage at which dental caries is likely to be treated restoratively [17].

1 $$ICER = Cost_{CWF} \;per\;d_{3vc} ft{/}D_{3vc} MFT\;Prevented_{CWF} = \frac{{Annual\;Cost\;of\;Supply_{CWF} }}{{Annual\;d_{3vc} ft{/}D_{3vc} \;MFT\;Prevented_{CWF} }}$$

### The annual cost to supply CWF

The costs covered by the state for the provision of CWF are the capital costs, the operating and maintenance (O&M) costs and the fluoride chemical cost (Eq. 2).

2 $$Annual\;Cost\;of\;Supply_{CWF} = Capital\;Cost_{CWF} + O\& M\;Cost_{CWF} + Fluoride\;Chemical\;Cost$$

The EPA provided information on the population served and the annual average daily throughput of treated water for each water treatment scheme (WTS) on the PWS delivering CWF in 2017 [12]. Capital and O&M costs were estimated from a national audit of the water fluoridation process [20] along with cost information provided by the Health Service Executive (HSE) to the authors. The national audit, conducted in 2008/09, identified each element where a WTS failed to comply with the Code of Practice [38] and outlined the capital investment and O&M costs required by the scheme to achieve compliance. To estimate such costs, the water treatment schemes were categorised according to their average annual throughput in m^3^/day (< 1000 to > 20,000 m^3^/day) corresponding to the categories reported by the audit [20]. Fluoride chemical costs were informed by the HSE’s contracted cost to the single supplier of Hydrofluosilicic acid (H_2_SiF_6_), the only chemical used to fluoridate the PWS in Ireland.

### Capital costs

Two forms of capital expenditure were identified: (i) planned capital expenditure, the costs to commence CWF, and (ii) reactive capital expenditure, the finance required to replace existing equipment (Eq. 3).

3 $$Capital\;Cost_{CWF} = Planned\;Capital\;Costs + Reactive \;Capital\;Costs$$

The costs reported by the national audit were taken as the planned capital costs per WTS category *‘i’* inflated to 2017 prices using the consumer price index [39] and depreciated over a 30-year time frame, the expected useful life of a WTS (Eq. 4). This depreciation rate may overestimate the cost of CWF, as many schemes have been operating for over fifty years without replacement.

4 $$Planned\;Capital\;Cost_{i} = \frac{{Cost\;WTS_{i} \left( {1 + inf_{2017} } \right)}}{{t_{dep} }}$$

The reactive expenditure was taken as the total accounting cost advised by the HSE for each WTS to maintain the required levels of fluoride in 2017. This cost was weighted according to the distribution of total planned capital expenditure per WTS category and divided by the number of schemes within each category to yield an annual reactive cost per WTS (see Eq. 5 where TC is the total outlay recorded by the HSE in 2017, *x*_*i*_ is the cost for the WTS category *i* reported by the national audit, and *y*_*i*_ is the number of schemes in category *i*). The depreciated planned capital cost and the annual reactive capital cost were summed to produce an annual capital cost per WTS category.

5 $$Reactive\;Capital\;Cost_{i} = TC\left( {\frac{{x_{i} }}{{\sum x_{i} *y_{i} }}} \right)$$

### The operating and maintenance costs

The annual O&M cost was assumed to be an average of the national audit cost adjusted for inflation [39] and the HSE reported O&M costs for each WTS category.

### The fluoride chemical cost

The annual fluoride chemical cost was calculated specific to each WTS, based on the volume of water produced, the required concentration of fluoride (0.7 mg/l) [40], the specific gravity of H_2_SiF_6_ and the HSE contracted cost per litre of H_2_SiF_6_ for 2017 (Eq. 6).

6 $$Fluoride\;Chemical\;Cost = m^{3}/day \left( {Cost\;H_{2} SiF_{6} \;per\;litre*\frac{365}{{1000}}*\frac{{mg\;F\;in\;water}}{{F\;per\;kg\;H_{2} SiF_{6} }}*\frac{1}{{specific\;gravity\;H_{2} SiF_{6} }} } \right)$$

At the end of the costing process, each WTS had an annual capital and O&M cost attributed according to its scheme category, along with a fluoride chemical cost specific to each WTS. The schemes were further classified according to the size of the population served (< 1000 to > 100,000 people). The total annual cost for each WTS category was divided by the population served to produce a mean annual per person cost per WTS category. Informed by national population statistics [41] and the populations served by each WTS [12], the total annual cost of CWF provision to 5-, 8-, and 12-year-old schoolchildren was calculated.

### Annual dental caries prevented

The dental caries prevented attributable to CWF was estimated using anonymized dental caries outcome data, recorded at the dentine level with or without cavitation (d_3vc_mft/D_3vc_MFT), from a representative sample of schoolchildren living in Ireland collected in the FACCT study between 2013 and 2017 [31]. This cross-sectional oral health survey with a longitudinal component, examined children aged 5- and 12-years in the 2013/14 school year and re-examined the 5-year-old children aged 8 in the 2016/17 school year. The analysis was confined to two categories of children: children with (‘fluoridated’) and children without (‘non-fluoridated’) lifetime exposure to CWF. This classification was made following detailed examination of the fluoridation status of the domestic water supply for each child’s current and past water supplies [31]. Information on the number of fluoridated and non-fluoridated schoolchildren sampled is provided in Table 1.

**Table 1:** Number of children clinically examined by age-group and lifetime exposure to CWF. *Source*: Author’s analysis of data from the FACCT Study [31]

Age-group (years)	Fluoridated	Non-fluoridated
5	1422	929
8	1080	772
12	946	644

The annual value for dental caries prevented owing to the intervention was calculated by combining the decay ($d_{3vc} ft{/}D_{3vc} FT$.) prevented by exposure to fluoride with the number of missing teeth prevented (Eq. 7).

7 $$Annual\;Caries (d_{3vc} ft{/}D_{3vc} MFT)\;Prevented _{CWF} = Annual\;Decay\;Prevented_{CWF} + Annual\;Missting\;Teeth\;Prevented_{CWF}$$

8 $$Annual\;Decay\;(d_{3vc} ft{/}D_{3vc} FT) \;Prevented _{CWF} = \left( {\frac{{d_{3vc} ft{/}D_{3vc} FT_{{nonF_{i} }} - d_{3vc} ft{/}D_{3vc} FT_{{nonF_{i - 1} }} }}{ years\;diff}*\left| {\frac{{d_{3vc} ft{/}D_{3vc} FT_{{F_{i} }} - d_{3vc} ft{/}D_{3vc} FT_{{nonF_{i} }} }}{{d_{3vc} ft{/}D_{3vc} FT_{{nonF_{i} }} }}} \right|} \right)$$

The decay prevented was estimated from the decay (d_3vc_ft/D_3vc_FT) increments for the non-fluoridated cohort (*nonF*) by age-group *‘i’,* where* i* takes the value 0, 1, 2 or 3 corresponding to 1-year-old, 5-year-old, 8-year-old, and 12-year-old children, respectively (first term Eq. 8). We did not have a baseline caries measure to calculate the caries increment for 5-year-old children, therefore it was assumed that the decay occurred between ages 1 and 5 based on the chronology of deciduous tooth eruption and expert opinion. The mean caries increments were further divided by the difference in the number of years between age-groups (years *diff*) to generate a mean annual caries increment specific to each age-group for the non-fluoridated children (first term Eq. 8).

To determine the decayed teeth (d_3vc_ft/D_3vc_FT) prevented attributable to one year of exposure to the intervention, the effect of CWF, calculated as the difference in the decay experience between the fluoridated and non-fluoridated cohorts for the three age-groups, was applied to the relevant annual decay increment for each group. This method assumed the effect of CWF was constant over time [24] and also controlled for exposure to other fluoride sources between the two cohorts (second term Eq. 8).

9 $$Annual \;Missing\;Teeth\;Prevented_{CWF} = \left( {\frac{{MT_{{nonF_{i} }} - MT_{{nonF_{i - 1} }} }}{years\;diff}*\left| {\frac{{MT_{{F_{i} }} - MT_{{nonF_{i} }} }}{{MT_{{nonF_{i} }} }}} \right|} \right)$$

The number of missing teeth prevented was confined to the permanent dentition of 12-year-old children as data on missing teeth for the 5- and 8-year-old age-groups are unreliable given the transition from primary to permanent teeth at these ages (Eq. 9).

### Net cost CWF per decayed, missing or filled tooth prevented

Thus far, the analysis had not considered the potential treatment savings associated with the caries prevented attributable to CWF. The incremental cost-effectiveness ratio was recalculated to include the potential treatment savings attributable to exposure to the CWF intervention in 2017 (Eq. 10).

10 $$ICER = Net\;Cost_{CWF} per d_{3vc} ft{/}D_{3vc} MFT\;Prevented_{CWF} = \frac{{Annual\;Cost_{CWF} - Annual\;Treatment\;savings_{{_{CWF} }} }}{{Annual\;d_{3vc} ft{/}D_{3vc} MFT\;Averted_{CWF} }}$$

### Annual treatment savings

The potential annual treatment savings attributable to CWF was estimated as the annual caries prevented attributable to CWF multiplied by the present value (PV) of the lifetime cost to treat the caries prevented during 2017 (Eq. 11).

11 $$Annual\;Treatment\;Savings _{CWF} = Annual\;d_{3vc} ft{/}D_{3vc} MFT\;Prevented_{CWF} * PV\;Lifetime\;Treatment\;Cost\;of\;Decay$$

### Lifetime treatment cost of decay

We hypothesized that all dental caries, whether treated or untreated, carried the full cost of treatment given the quality of life benefits associated with good oral health [22–24, 30, 42]. Exposure to CWF confers a continuous benefit as once the tooth structure is damaged it requires restoration and follow-up maintenance throughout life. Thus, the initial and expected follow-up treatments required to maintain a carious tooth over a lifetime were considered (Eq. 12).

12 $$PV\;Lifetime\;Treatment\;Cost\;of\;Caries = PV\left( {Cost\;Initial\;Tretament + Cost\;Subsequent\;Treatments} \right)$$

In Ireland, oral healthcare is delivered through a two-tiered system of public and private services. Citizens who qualify for the means tested medical card are entitled to a limited range of publicly funded treatments, whilst all other persons pay the full cost for treatment [43]. For this analysis, we assumed all treatments were delivered in the primary care setting and treatment occurred at the end of 2017.

The initial treatment types were limited to a restoration for the 5- and 8-year-old age-groups, and a restoration or an extraction for the 12-year-old age-group. Further to the EU’s ratification of the Minamata Convention on Mercury [44], initial restorations occurred under the minimum age required for a dental amalgam and were assumed to be composite restorations [45]. The number of initial treatments were estimated for each age-group according to the number of dental caries prevented (Eq. 8). Extractions for the 12-year-olds, estimated as per the number of missing teeth prevented (Eq. 9), were allocated between routine and surgical according to the distribution of extractions in the public system for the youngest age cohort.

In the absence of longitudinal treatment information, treatments delivered by the publicly funded system during 2017/18 served as a proxy for the distribution of expected follow-up treatment types for both publicly and privately funded individuals [46]. Follow-up treatments applied to the permanent dentition of the 8- and 12-year-old age-groups only and were limited to replacement fillings and extractions. The expected longevity of a restoration was assumed to be 12 years [22–24, 47, 48] and replacement restorations were assumed to fail at the same rate as initial restorations [24]. Restorative crowns were not included as they are not funded under the public system.

The probabilistic model simulated each age-group through the possible treatment cycles from initial treatment until death at the average life expectancy at 82 years [24, 49]. The range of expected follow-up treatments delivered at each cycle, were conditional on the treatment received in the preceding cycle, the age at the likely time of successive treatment, the probability of the follow-up treatment type weighted according to the publicly funded treatment distribution [50], the probability of survival to the time of the follow-up treatment [49], and life expectancy [49]. The public system’s treatment distribution accounted for the possibility that the tooth would be extracted during the lifetime treatment of the carious tooth. The probability of receiving a particular treatment *i* at treatment cycle *T,* given the treatment type received in the preceding cycle, was assumed to be independent of treatment type^2^ (Eq. 13).

13 $$Prob\left( {Treatment_{i, T} } \right) = \mathop \sum \limits_{j = 1}^{4} \mathop \sum \limits_{i = 1}^{4} Prob\left( {Treatment_{i, T} } \right)Prob\left( {Treatment_{j, T - 12} } \right)$$

A treatment cost was assigned to each treatment type at each cycle. Public treatment costs were taken from the 2017 HSE report on treatment expenditures [50], and private costs were collected from a representative sample of dental practices in 2017. All treatment costs were discounted at 4% per annum [34, 35]. An expected discounted lifetime treatment cost was derived for each age-group for both public and private treatments (Eq. 14) where *n* is the number of restorations a patient undergoes over a lifetime of dental care.

14 $$PV\left( {Cost\;Initial\;Treatment + Cost\;Subsequent\;Treatments} \right) = \mathop \sum \limits_{k = 0}^{n} \mathop \sum \limits_{i = 1}^{4} \frac{{Prob\left( {Treatment_{i} } \right)*Cost\left( {Treatment_{i} } \right)}}{{\left( {1 + discount\;rate} \right)^{1 + k12} }}$$

The likely indirect savings accruing to CWF, in terms of productivity losses averted, were also valued. Further to expert opinion, one hour in lost productivity per treatment was assumed. The 2017 average hourly total labour cost of €26.01 [51] was added to each treatment cost and modelled as above.

The lifetime treatment costs were multiplied by the age specific annual caries increments to estimate the potential annual lifetime treatment savings owing to one year of exposure to CWF. These treatment savings were weighted according to the proportion of publicly (33%) and privately (67%) funded individuals in 2017 [52] to produce a single annual lifetime treatment saving per age-group.

Information on the treatment probabilities, survival probabilities at each treatment cycle, the costs of dental treatments along with a sample calculation of an expected follow up treatment are available in Additional file 1: Tables S1–S3 and Sample Calculation 

### Accounting for uncertainty

One-way sensitivity analysis considered the impact of the discount rate, CWF efficacy, treatment costs, treatment longevity, CWF costs and the depreciation rate. Scenario analyses varied these parameters simultaneously under a worst- and best-case combination of input values. The parameters varied along with their input values (best-, worst-case) were the discount rate (0%, 10%), different levels of CWF effectiveness (+ 20%, − 20%), the potential treatment savings (+ 20%, − 20%), the lifetime of a restoration (10 years, 15 years), CWF operating and maintenance costs (+ 20%, − 20%) and the depreciation rate for CWF capital costs (50 years, 10 years). The probabilistic sensitivity analyses (PSA) evaluated the combined parameter uncertainty in the study’s input values using a 10,000 iteration Monte Carlo simulation. An overview of parameters varied in the probabilistic sensitivity analysis are available in Additional file 1: Table S4.

### Dental fluorosis

Previous reviews of economic evaluations of CWF encourage the inclusion of treatment costs resultant from enamel fluorosis in CWF cost estimates [53, 54]. In the absence of published estimates, the cost to treat fluorosis was not included in the formal CEA. However, using information on the prevalence of fluorosis according to lifetime fluoridation status, collected in the FACCT study [31] as outlined in Table 2, the per capita treatment cost required to negate the potential treatment savings attributable to CWF were estimated.

**Table 2:** Dean’s Index of Fluorosis, percentage of children affected according to age-group and fluoridation status. *Source*: Author’s analysis of data from the FACCT Study [31]

Age group (years)	8		12	
Dean’s Index of Fluorosis	Fluoridated	Non-fluoridated	Fluoridated	Non-fluoridated
Normal%	62.66	79.27	63.58	82.46
Questionable%	21.51	15.54	23.29	13.61
Very mild%	12.38	4.92	9.71	3.28
Mild%	3.17	0.26	2.87	0.66
Moderate%	0.28	0.00	0.55	0.00

## Results

The per capita annual cost of CWF provision was €2.15 pp, or a total cost of €7.3 million to the Irish state. Similar to findings from other jurisdictions [22, 23, 30, 53], economies of scale were observed, with costs ranging between €0.54 pp to €39.19 pp for serving populations > 100,000 and < 1000 respectively. Table 3 outlines the average per capita cost of CWF according to the WTS population size served and age group. Informed by national population statistics and the populations served by each WTS, the total cost of CWF provision to 5-, 8-, and 12-year-old schoolchildren (n = 148,910) was estimated at €320,664 in 2017.

**Table 3:** The cost of CWF provision according to WTP population size served and age group. *Source*: Authors analysis of data provided by the Environmental Protection Agency [12], CSO [41], national audit of the CWF process [20] and the HSE

WTP size	< 1000	1000–4999	5000–19,999	20,000–99,999	> 100,000	Total
CWF population served %	0.45	7.73	21.38	23.39	47.05	100
Per capita cost CWF €	39.18	9.38	3.16	1.38	0.54	2.15
Total cost CWF provision € 5 years	9176.53	37,561.20	35,017.06	16,657.36	13,074.26	111,486.41
	*(n* = *234)*	*(n* = *4004)*	*(n* = *11,067)*	*(n* = *12,109)*	*(n* = *24,359)*	*(n* = *51,772)*
Total cost CWF provision € 8 years	9,065.00	37,104.68	34,591.46	16,454.91	12,915.35	110,131.40
	*(n* = *231)*	*(n* = *3955)*	*(n* = *10,932)*	*(n* = *11,961)*	*(n* = *24,063)*	*(51,143)*
Total cost CWF provision € 12 years	8,152.59	33,370.04	31,109.78	14,798.70	11,615.40	99,046.51
	*(n* = *208)*	*(n* = *3557)*	*(n* = *9832)*	*(n* = *10,758)*	*(n* = *21,641)*	*(45,995)*

### Annual dental caries prevented

The average caries level by presence of CWF, the effectiveness of CWF and the estimated mean caries increments prevented in 2017 specific to each age group are presented in Table 4.

**Table 4:** Average caries levels by presence of CWF, effectiveness CWF and caries prevented attributable to CWF. *Source*: Authors analysis of data from the FACCT Study [31], d_3vc_ft/D_3vc_ MFT data rounded to two decimal places

Age group	Average caries levels						CWF effectiveness			Annual caries prevented		
	With CWF			Without CWF			%					
Years	d_3vc_ft	D_3vc_FT	MT	d_3vc_ft	D_3vc_FT	MT	d_3vc_ft	D_3vc_FT	MT	d_3vc_ft	D_3vc_FT	MT
5	0.96	–	–	1.72	–	–	44	–	–	0.19	–	–
8	1.69	0.26	–	2.30	0.37	–	27	29	–	0.11	0.03	–
12	–	0.71	0.04	–	1.32	0.07	–	46	45	–	0.11	0.01

### The cost of CWF provision per decayed, missing or filled tooth prevented

The incremental cost per d_3vc_ft/D_3vc_ MFT prevented in 2017 attributable to CWF was €14.09. Table 5 provides data on the total d_3vc_ft/D_3vc_ MFT prevented along with the incremental cost per d_3vc_ft/D_3vc_ MFT prevented according to WTS population size served and age group.

**Table 5:** Total number and incremental cost per d_3vc_ft/D_3vc_ MFT prevented. *Source*: Authors analysis of data from the FACCT Study [31] the Environmental Protection Agency [12], CSO [41], national audit of the CWF process [20] and the HSE. A positive value indicates a net cost and d_3vc_ft/D_3vc_ MFT data is rounded to two decimal places

	WTP population size					
	< 1000	1000–4999	5000–19,999	20,000–99,999	> 100,000	Total
5 years						
Total number d_3vc_ft averted	44.51	760.84	2103.15	2301.16	4629.25	9838.90
Cost per mean d_3vc_ft averted €	206.19	49.37	16.65	7.24	2.82	11.33
8 years						
Total number d_3vc_ft/D_3vc_ FT averted	34.24	585.34	1618.02	1770.36	3561.44	7569.40
Cost per mean d_3vc_ft/D_3vc_ FT averted €	264.75	63.39	21.38	9.29	3.63	14.55
12 years						
Total number D_3vc_ MFT averted	24.20	413.74	1143.69	1251.37	2517.37	5350.37
Cost per mean D_3vc_ MFT averted €	336.86	80.65	27.20	11.83	4.61	18.51

### Annual treatment savings

The potential annual treatment savings (direct and indirect) associated with caries prevented for 5-, 8-, and 12-year-old schoolchildern was €16.33, €17.89, and €25.94 respectively, or a total saving of €2.95 million to the health-payer, of which 71% related to direct treatment savings, which is slightly higher than the western European average of 64% [6].

### Net cost of CWF

The net cost of CWF (cost of CWF provision minus the expected treatment savings associated with dental caries prevented) for 5-, 8-, and 12-year-old schoolchildren was estimated at -€2.63 million in 2017, or an average cost of -€17.68 per child, with negative costs representing a cost saving to the health-payer. For the schemes serving populations < 1000, the cost of supply outweighed the likely treatment savings for all age cohorts. However, for schemes serving communities > 1000, cost savings were present as outlined in Table 6. Given the lesser proportion of the fluoridated population served by smaller schemes, the mean per capita cost across all age-groups was negative, representing an overall saving to the health-payer and a return on investment ((CWF provision costs – treatment savings/CWF costs) *100) estimated at 820%

**Table 6:** Mean per capita net cost CWF and incremental cost (including potential treatment savings) per d_3vc_ft/D_3vc_MFT prevented. *Source*: Authors analysis of data from the FACCT Study [31] the Environmental Protection Agency [12], CSO [41], national audit of the CWF process [20] and the HSE

	WTP size	< 1000	1000–4999	5000–19,999	20,000–99,999	> 100,000	Average	PSA
Net cost CWF (€)*	5 years	22.86	− 6.95	− 13.16	− 14.95	− 15.79	− 14.18	− 13.75 SD: 3.67 UI: [− 8.09 to − 20.14]
	8 years	21.30	− 8.51	− 14.72	− 16.51	− 17.35	− 15.73	− 15.42 SD: 3.04 UI: [− 10.62 to − 20.59]
	12 years	13.25	− 16.55	− 22.77	− 24.56	− 25.40	− 23.78	− 23.85 SD: 5.10 UI: [− 15.53 to − 32.24]
Net cost per d_3vc_ft/D_3vc_MFT prevented in 2017 (€)*	5 years	120.26	− 36.56	− 69.27	− 78.68	− 83.10	− 74.59	− 72.35 SD:19.31 UI: [− 42.57 to − 105.98]
	8 years	143.89	− 57.47	− 99.48	− 111.56	− 117.23	− 106.31	− 104.19 SD: 20.54 UI: [− 71.75 to − 139.12]
	12 years	113.90	− 142.30	− 195.76	− 211.13	− 218.34	− 204.44	− 205.03 SD: 43.84 UI: [− 133.51 to − 277.16]

*A negative value indicates a net cost saving, a positive value indicates a net cost

### Net cost CWF per decayed, missing or filled tooth prevented

The ICER, or the net cost per d_3vc_ft/D_3vc_ MFT prevented in 2017 for schoolchildren was estimated at -€115.67, indicating a cost saving to the health-payer and a positive return on investment. Table 6 provides the net cost per d_3vc_ft/D_3vc_MFT prevented in 2017 according to WTS size and age group.

### Sensitivity analysis

One-way sensitivity analysis confirmed the study results were most sensitive to assumptions around the discount rate, CWF efficacy and the lifetime cost of dental treatment. Scenario analysis revealed that, even under worst-case conditions, when treatment savings were included CWF yielded negative net costs per d_3vc_ft/D_3vc_MFT prevented. The parameters varied along with the resultant average net cost per d_3vc_ft/D_3vc_MFT prevented in 2017 are outlined in Table 7.

**Table 7:** Overview of parameters varied in the scenario analysis and results of the analyses

Parameter	Best-case%	Reference-case%	Worst-case%
Scenario analysis			
Discount rate	0	4	10
CWF efficacy	+ 20	100	− 20
Treatment costs	+ 20	100	− 20
Treatment life (years)	10 years	12 years	15 years
CWF cost	− 20	100	+ 20
Depreciation (years)	50 years	30 years	10 years

	Best-case	Reference-case	Worst-case
Incremental cost (including potential treatment savings) per d_3vc_ft/D_3vc_MFT prevented €*			
5 years	− 99.30	− 74.59	− 46.95
8 years	− 200.70	− 106.31	− 52.07
12 years	− 502.93	− 204.44	− 77.50
Total	− 227.96	− 115.67	− 55.84

*A negative value indicates a net cost saving

The probabilistic sensitivity analysis reported the mean net cost of CWF per d_3vc_ft/D_3vc_MFT prevented was -€72.35 (SD: €19.31, UI: -€42.57 to -€105.98) per 5-year-old child, -€104.19 (SD: €20.54, U: -€71.75 to -€139.12) per 8-year-old child, and -€205.03 (SD: €43.84, UI: -€133.51 to -€277.16) per 12-year-old child. Using per capita point estimate values leads to an expected national net annual CWF cost of -€2.59 million for all 5-, 8-, and 12-year-old children with lifetime exposure to CWF (mean -€17.44 per child), or a cost of—€114.13 per d_3vc_ft/D_3vc_MFT prevented in 2017. The values reported by the PSA are presented in Table 6.

### Dental fluorosis

The international consensus around the aesthetic perception of fluorosis considers cases with Dean’s “mild” or “more” of concern [55]. Extrapolating the prevalence of “mild” and “moderate” cases in FACCT to national population estimates of 8- and 12-year-old schoolchildren with lifetime exposure to CWF, an annual fluorosis treatment cost of €414 per affected child was required to negate the treatment savings attributable to CWF. When the “moderate” grade fluorosis category was considered in isolation, the annual per capita cost to treat fluorosis would need to be more than €3483 per affected child to render the treatment savings to zero for both age-groups.

## Discussion

The findings from our analysis show a variation in the returns on investment in CWF, with larger populations benefiting from economies of scale, a common observation of previous research [21–26, 30, 53]. In 2017, the incremental cost per d_3vc_ft/D_3vc_ MFT prevented for 5-, 8-, and 12-year-old schoolchildren increased across all age groups as the population of the community served by the WTS decreased. Across all WTS populations served and the three age groups, the incremental cost per d_3vc_ft/D_3vc_ MFT prevented was €14.09. Whether this cost is deemed an appropriate allocation of public resources depends on how oral health is valued by society. That said, this cost did not consider the likely savings associated with the reduced need for dental treatments, but, given the theory that oral health care systems are supply driven with treatment savings being replaced by other services or higher fees to maintain incomes [21, 56], it is important these costs are highlighted.

The aim of this study was to provide evidence to inform policy makers on the continued public provision of CWF. However, should the Irish system choose to discontinue its CWF programme, the oral healthcare requirements of the population in receipt of CWF would certainly expand due to increased caries levels.

When the potential treatment savings associated with the caries prevented for the 5-, 8-, and 12-year-old schoolchildren were included in the analysis, all fluoridated schemes serving populations greater than 1000 provided cost savings to the health-payer. The incremental cost per d_3vc_ft/D_3vc_ MFT prevented, for the three age groups across all the WTS populations served, was estimated at -€115.67. The results of this analysis were robust to both deterministic and probabilistic sensitivity analyses. The PSA, which stochastically varied CWF efficacy, dental treatment costs and the cost of CWF provision, produced similar results, thus supporting a positive return on investment for CWF.

It is important to note that the estimates around the expected lifetime treatment costs may have undervalued the potential savings attributable to CWF for these schoolchildren. Firstly, in the absence of longitudinal treatment information, publicly funded treatments, delivered to individuals between 16 and 75+ years across a two-year period, were used as a proxy for the follow-up treatments over a lifetime for both publicly and privately funded persons. This was a crude process to derive treatment probabilities as it assumed that all teeth beyond a failed filling were extracted, which negatively biased the cost-effectiveness of CWF. Also, the limited treatment range disregarded consumer choice and the broad gamut of services available to individuals within the private system. For example, a written communication from the principal dental insurer, showed that in 2013, 13% of subsidized restorations were crowns, the cost of which was estimated at nearly seven times that of a composite restoration. Whilst we acknowledge consumption of health services differ between those paying out of pocket and the insured, exclusion of this treatment type from the analysis is not reflective of current practice. Furthermore, confining the analysis to the primary healthcare setting overlooks the more complex and costly procedures delivered by secondary and specialist healthcare services.

Thirdly, assumptions around a single failure rate for initial and follow-up restorations, along with an equal cost for both single and multi-surface restorations, did not allow for the possibility of treatments between designated treatment cycles, or for the complexity involved with the continued replacement of restorations. And finally, the indirect treatment cost did not incorporate the complete financial burden associated with absenteeism from school and work due to dental disease. In consideration of these facts, the estimated lifetime treatment savings reported are conservative and almost certainly underestimate the potential savings delivered by CWF.

We recognise that the benefits of CWF, in terms of the dental caries prevented, are not exclusive to the age-groups included in this analysis. Future research should consider the impact of CWF across all age-groups and community sizes in Ireland.

## Conclusion

The economic evidence, provided by this analysis, shows that the overall benefits returned by CWF exceed the total cost of providing the intervention for these schoolchildren. CWF is a universal intervention to ensure acceptable levels of fluoride exposure for all, irrespective of income or social circumstances. Based on the current data available on the effectiveness and cost of CWF, dental caries levels, dental treatment costs, and fluorosis, there are strong economic grounds for the continued public provision of CWF for these schoolchildren despite the current environment of multiple fluoride sources. CWF as a health intervention provides a reduction in the national disease burden and offers cost-savings to the health-payer, which in many jurisdictions is often the private individual.

## Supplementary Information

**Additional file 1: Supplementary Table 1**. Treatment probabilities. **Supplementary Table 2**. Survival probabilities. **Supplementary Table 3** Dental treatment costs. **Sample Calculation** Expected follow-up treatment sample calculation. **Supplementary Table 4** Overview of parameters varied in the probabilistic sensitivity analysis. **Supplementary Table 5**. Overview of study parameters in the reference case **Supplementary Table 6**. CHEERS checklist.

## Data Availability

The datasets used and analysed during the current study are available from the corresponding author on request.

## Abbreviations

CWF: Community water fluoridation
PWS: Public water supply
EPA: Environmental Protection Agency Ireland
WTS: Water treatment scheme
HSE: Health Service Executive
FACCT study: Fluoride and Caring for Children’s Teeth
O&M: Operating and maintenance
H_2_SiF_6_: Hydrofluosilicic acid
d_3vc_mft/D_3vc_MFT: Decayed, missing or filled teeth recorded at the dentine level, with or without cavitation
PSA: Probabilistic sensitivity analysis

## Footnotes

1 adjusted to EU19 prices (5).

2 The probability of receiving an amalgam given that you received an extraction at your previous visit is of course zero.
