# Supplementary material for: A cost-effectiveness analysis of community water fluoridation for schoolchildren
Source: BMC Oral Health. 2021 Mar 25;21:158. doi: 10.1186/s12903-021-01490-7 (PMC7995596; doi:10.1186/s12903-021-01490-7)
Supplement: Supplementary file 1 — Additional file 1: Supplementary Table 1. Treatment probabilities. Supplementary Table 2. Survival probabilities. Supplementary Table 3 Dental treatment costs. Sample Calculation Expected follow-up treatment sample calculation. Supplementary Table 4 Overview of parameters varied in the probabilistic sensitivity analysis. Supplementary Table 5. Overview of study parameters in the reference case Supplementary Table 6. CHEERS checklist. [file 12903_2021_1490_MOESM1_ESM.docx]

**Additional file 1**

This document provides additional information in support of items referred to in the main manuscript. Tables 1-3 present the treatment probabilities (1), survival probabilities (2) and cost of dental treatments (3) that was used for the calculation of reference case expected future follow-up treatment savings. This is followed by an early follow up replacement restoration calculation. Table 4 presents an overview of the parameters varied in the probabilistic sensitivity analysis and Table 5 outlines the parameters employed by the reference-case. Table 6 and provides the completed CHEERS checklist (4).

**Additional file 1: Table S1**

**Treatment probabilities** (1)

| **Treatment numbers** | | | | | | | | |
| --- | --- | --- | --- | --- | --- | --- | --- | --- |
| **Age group** | **16-24** | **25-34** | **35-44** | **45-54** | **55-64** | **65-69** | **70-74** | **>75** |
| **Amalgam** | 68,582 | 75,249 | 88,158 | 79,581 | 59,769 | 25,489 | 25,371 | 30,488 |
| **Composite** | 16,586 | 23,364 | 35,037 | 40,860 | 44,956 | 26,296 | 29,702 | 43,058 |
| **Routine** | 16,488 | 25,598 | 36,964 | 48,088 | 49,787 | 22,944 | 23,339 | 32,402 |
| **Surgical** | 7,709 | 13,842 | 15,862 | 14,455 | 11,953 | 5,307 | 5,478 | 8,302 |
| **Total** | **109,365** | **138,053** | **176,021** | **182,984** | **166,465** | **80,036** | **83,890** | **114,250** |
| **Treatment proportions** | | | | | | | | |
| **Amalgam** | 62.7% | 54.5% | 50.1% | 43.5% | 35.9% | 31.8% | 30.2% | 26.7% |
| **Composite** | 15.2% | 16.9% | 19.9% | 22.3% | 27.0% | 32.9% | 35.4% | 37.7% |
| **Routine** | 15.1% | 18.5% | 21.0% | 26.3% | 29.9% | 28.7% | 27.8% | 28.4% |
| **Surgical** | 7.0% | 10.0% | 9.0% | 7.9% | 7.2% | 6.6% | 6.5% | 7.3% |

Source: HSE Primary Care Reimbursement Service. Statistical Analysis of Claims and Payments 2017. [Internet]. 2017. Available from: <https://www.hse.ie/eng/staff/pcrs/pcrs-publications/annual-report-2017.pdf>

**Additional file 1: Table S2**

**Survival probabilities** (2)

| **Probability of being alive at age i given that you were alive for the previous restoration** | | |
| --- | --- | --- |
| **age i** | **1^st^ treatment at age 9** | **1^st^ treatment at age 13** |
| 9 |  | 99.99% |
| 13 | 99.99% |  |
| 21 |  | 99.74% |
| 25 | 99.53% |  |
| 33 |  | 99.32% |
| 37 | 99.25% |  |
| 45 |  | 98.76% |
| 49 | 98.32% |  |
| 57 |  | 96.73% |
| 61 | 94.96% |  |
| 69 |  | 90.25% |
| 73 | 85.71% |  |

Source: Central Statistics Office, Ireland. Irish Life Tables No.16, 2010-2012 [Internet]. Available from: <https://pdf.cso.ie/www/pdf/20171121010152_Irish_Life_Tables_No_16_20102012_full.pdf>

**Additional file 1: Table S3**

**Dental treatment costs** (3)

| **Treatment cost** | | |
| --- | --- | --- |
|  | **Public** | **Private** |
| **Amalgam** | €50.06 | €93.85 |
| **Composite** | €51.88 | €109.83 |
| **Routine** | €39.50 | €95.85 |
| **Surgical** | €70.00 | €161.08 |

Source: Public costs - HSE Primary Care Reimbursement Service. Statistical Analysis of Claims and Payments 2017. [Internet]. 2017. Available from: <https://www.hse.ie/eng/staff/pcrs/pcrs-publications/annual-report-2017.pdf>. Private costs – authors survey of private dental treatment costs in Ireland in 2017.

**Supplementary Information**

**Expected follow-up treatment sample calculation.**

A 12-year-old patient in receipt of dental care from the public system, that prevented a composite filling at age 13, having had one year of exposure to CWF in 2017, would realise potential savings according to the following calculation. The present value of the first year’s dental care is €49.88 which discounts the cost of a composite filling on the public system by the one-year discount rate and adjust for the 1-year probability of mortality between ages 12 and 13.

$$€49.88={1.04}^{-1}*€51.88*0.999$$

Given an assumed restoration life of 12 years, the present value of the second treatment prevented would be €30.13 which again discounted the weighted cost of the treatment at age 25. Weights were taken from the treatment distribution in Table 1 and a simplifying assumption of independence between procedures was made. This figure was adjusted for the mortality of the 25-year-old given that the patient was alive at age 13 (Table 2).

$$€30.13={1.04}^{-13}*\left( €50.06*0.545+ €51.88*0.169+€39.50*0.185+€70*0.1 \right)*0.9953$$

Subsequent restoration costs averted were calculated in a similar fashion and the resulting summation gives the present value of potential treatment costs averted.

**Additional file 1: Table S4**

**Overview of parameters varied in the probabilistic sensitivity analysis**

| **Parameter** | **Mean** | **SD** | **Distributional assumption** |
| --- | --- | --- | --- |
| CWF effectiveness | observed mean | +/- 20% | Normal, truncated at 0 and 1 |
| **Treatment savings** | | | |
| Public treatments | observed mean | +/- 20% | Lognormal |
| Private treatments | observed mean |  | Gamma - shape and scale parameters chosen to fit survey data using Kolmogorov-Smirnov statistics |
| Indirect treatments | observed mean | +/- 20% | Normal, left skew parameter 0.2 |
| **CWF Costs** | | | |
| Mean pp costs | observed mean | +/- 20% | Lognormal |
| Costs WWTP size | observed mean | +/- 20% | Lognormal |

**Additional file 1: Table S5. Overview of study parameters in the reference case**

| **Parameters** | **Values** | | **Source** |
| --- | --- | --- | --- |
| **CWF Supply Costs** |  | |  |
| Population served by WTS | Study population = 148,910 | | Published Data |
| CWF per capita cost by size of community served | | | |
| *<1,000* | €39.18 | | Authors analysis of data from published sources (See main manuscript - annual cost to supply CWF and Table 3) |
| *1,000 - 4,999* | €9.38 | |  |
| *5,000 - 19,999* | €3.16 | |  |
| *20,000 - 99,000* | €1.38 | |  |
| *>100,000* | €0.54 | |  |
| **Annual Caries Prevented** | | | |
| Caries levels | With CWF | Without CWF | Authors analysis of data from FACCT |
| *5 yrs (d3vcft)* | 0.96 | 1.72 |  |
| *8 yrs (d3vcft/D3vcFT)* | 1.69/0.26 | 2.30/0.37 |  |
| *12 yrs (D3vcMFT)* | 0.75 | 1.39 |  |
| CWF Effectiveness |  |  | Authors analysis of data from FACCT |
| *5 yrs (d3vcft)* | 44% | |  |
| *8 yrs (d3vcft/D3vcFT)* | 27%/29% | |  |
| *12 yrs (D3vcMFT)* | 46% | |  |
| **Treatment Savings** | | | |
| Replacement rate | 12 years | | Published data |
| Treatment probabilities |  | | |
| *Amalgam* | age dependent, ranging between ( 26.7% to 62.7% to) | | HSE Distribution of Treatments - published data (Supplementary Table 1, Additional File 1) |
| *Composite* | age dependent, ranging between (15.2% to 37.7%) | |  |
| *Routine* | age dependent, ranging between (15.1% to 29.9%) | |  |
| *Surgical* | age dependent, ranging between (6.6% to 10.0%) | |  |
| Survival probabilities | age dependent, ranging between (6.6% to 10.0%) | | Published data (Supplementary Table 2, Additional File 1) |
| Life Expectancy | 82 years | | Published data |

**Additional file 1: Table S6**

CHEERS checklist (4)

| Section/item | Item no. | Recommendation | Reported on page no./line no. |
| --- | --- | --- | --- |
| Title and abstract |  |  |  |
| Title | 1 | Identify the study as an economic evaluation, or use more specific terms such as ‘‘cost-effectiveness analysis’’ and describe the interventions compared. | Title |
| Abstract | 2 | Provide a structured summary of objectives, perspective, setting, methods (including study design and inputs), results (including base-case and uncertainty analyses), and conclusions. | Objective: page 2, lines 22-23  Perspective: page 2, line 37  Setting: page 2, line 28  Methods: page 2, lines 29-44  Results: page 2, lines 47 – page 3, line 5  Conclusion: page 3, lines 8-14  **Revised version (RV)– see abstract** |
| Introduction |  |  |  |
| Background & objectives | 3 | Provide an explicit statement of the broader context for the study. | Page 4, lines 55-60  Page 5, lines 1-22  **RV– page 4 last paragraph, page 5 paragraph 1** |
|  |  | Present the study question and its relevance for health policy or practice decisions. | Page 5, lines 25-52  **RV– page 5 last paragraph, and continued page 6** |
| Methods |  |  |  |
| Target population and  subgroups | 4 | Describe characteristics of the base-case population and subgroups analyzed including why they were chosen. | Page 5, lines 47-52  Page 6, lines 5-9  **RV– page 6 page 7 – methods paragraph 1** |
| Setting and location | 5 | State relevant aspects of the system(s) in which the decision(s) need(s) to be made. | Page 6, lines 10-12  **RV –page 7 – methods paragraph 1** |
| Study perspective | 6 | Describe the perspective of the study and relate this to the costs being evaluated. | Page 6, line 17  **RV –page 7 – methods paragraph 1** |
| Comparators | 7 | Describe the interventions or strategies being compared and state why they were chosen. | Page 6, line 9-12  **RV –page 7 – methods paragraph 1** |
| Time horizon | 8 | State the time horizon(s) over which costs and consequences are being evaluated and say why appropriate. | Page 6, lines 18-24  **RV –page 7 – methods paragraph 1** |
| Discount rate | 9 | Report the choice of discount rate(s) used for costs and outcomes and say why appropriate. | Page6, lines 17-18  **RV –page 7 – methods paragraph 1** |
| Choice of health outcomes | 10 | Describe what outcomes were used as the measure(s) of benefit in the evaluation and their relevance for the type of analysis performed. | Page 6, line 34  **RV – Page 7 – methods – last paragraph** |
| Measurement of effectiveness | 11a | Single study–based estimates: Describe fully the design features of the single effectiveness study and why the single study was a sufficient source of clinical effectiveness data. | Page 9, lines 41-58  Page 10, 1-60  Page 11, lines 1-3  **RV – Pages 10, paragraph 4 to page 12, paragraph 2** |
|  | 11b | Synthesis-based estimates: Describe fully the methods used for the  identification of included studies and synthesis of clinical  effectiveness data. | N/A |
| Measurement and valuation of  preference-based outcomes | 12 | If applicable, describe the population and methods used to elicit preferences for outcomes. | N/A |
| Estimating resources and costs | 13a | Single study–based economic evaluation: Describe approaches used to estimate resource use associated with the alternative interventions. Describe primary or secondary research methods for valuing each resource item in terms of its unit cost. Describe any adjustments made to approximate to opportunity costs. | Page 7, lines 1-60  Page 8, lines 1-46  Page 9, lines 1-35  Page 12, lines 1-60  Page 13, lines 1-60  Page 14, lines 1-26  **RV – Pages 8 to page 10, paragraph 3 and Pages 13 - 16** |
|  | 13b | Model-based economic evaluation: Describe approaches and data sources used to estimate resource use associated with model health states. Describe primary or secondary research methods for valuing each  resource item in terms of its unit cost. Describe any adjustments  made to approximate to opportunity costs. | N/A |
| Currency, price date, and  conversion | 14 | Report the dates of the estimated resource quantities and unit costs.  Describe methods for adjusting estimated unit costs to the year of  reported costs if necessary. Describe methods for converting costs  into a common currency base and the exchange rate. | Page 2, line 39  Page 6, lines 12 &24  Page 7, line 59  Page 8, line 46  **RV – page 2, paragraph 2, page 7 paragraph 1, page 9 paragraph 2** |
| Choice of model | 15 | Describe and give reasons for the specific type of decision-analytic  model used. Providing a figure to show model structure is strongly  recommended. | Added text –  Page 2, line 40-44  Page 6, lines 13-18  **RV – page 2, paragraph 2**  **Page 7 paragraph 1**  **Appendix sample calculation** |
| Assumptions | 16 | Describe all structural or other assumptions underpinning the  decision-analytic model. | Page 12, lines 52-60  Page 13, line 1-28  **RV – page 14, paragraphs 1 and 2** |
| Analytic methods | 17 | Describe all analytic methods supporting the evaluation. This could  include methods for dealing with skewed, missing, or censored data;  extrapolation methods; methods for pooling data; approaches to  validate or make adjustments (e.g., half-cycle corrections) to a  model; and methods for handling population heterogeneity and  uncertainty. | Page 13, lines 30-48  **RV – page 14, paragraphs 3 – page 19 paragraph 1.**  **Supplementary Tables 1-3, Additional File 1** |
| Results |  |  |  |
| Study parameters | 18 | Report the values, ranges, references, and if used, probability  distributions for all parameters. Report reasons or sources for  distributions used to represent uncertainty where appropriate.  Providing a table to show the input values is strongly  recommended. | **RV - Supplementary Tables 4 and 5, Additional file 1** |
| Incremental costs and outcomes | 19 | For each intervention, report mean values for the main categories of  estimated costs and outcomes of interest, as well as mean  differences between the comparator groups. If applicable, report  incremental cost-effectiveness ratios. | **RV - Pages 18-19**  **Tables 3 - 6** |
| Characterizing uncertainty | 20a | Single study–based economic evaluation: Describe the effects of sampling  uncertainty for estimated incremental cost, incremental  effectiveness, and incremental cost-effectiveness, together with  the impact of methodological assumptions (such as discount rate,  study perspective). | **RV – Page 16 and Pages 19 – 2.**  **RV-Table 7 and Supplementary Table 4, Additional File 1** |
|  | 20b | Model-based economic evaluation: Describe the effects on the results of  uncertainty for all input parameters, and uncertainty related to the  structure of the model and assumptions. | N/A |
| Characterizing heterogeneity | 21 | If applicable, report differences in costs, outcomes, or cost effectiveness  that can be explained by variations between subgroups of patients with different baseline characteristics or  other observed variability in effects that are not reducible by more  information. | N/A |
| Discussion |  |  |  |
| Study findings, limitations,  generalizability, and current  knowledge | 22 | Summarize key study findings and describe how they support the  conclusions reached. Discuss limitations and the generalizability of  the findings and how the findings fit with current knowledge. | Page 18 + 19  **RV - Pages 21 +22** |
| Other |  |  |  |
| Source of funding | 23 | Describe how the study was funded and the role of the funder in the  identification, design, conduct, and reporting of the analysis.  Describe other nonmonetary sources of support. | Page 23, line 55-54  **RV – Page 25** |
| Conflicts of interest | 24 | Describe any potential for conflict of interest among study  contributors in accordance with journal policy. In the absence of a  journal policy, we recommend authors comply with International  Committee of Medical Journal Editors’ recommendations. | Page 23, lines 34-36  **RV – Page 25** |

**REFERENCES**

1. HSE Primary Care Reimbursement Service. Statistical Analysis of Claims and Payments 2017. [Internet]. 2017. Available from: https://www.hse.ie/eng/staff/pcrs/pcrs-publications/annual-report-2017.pdf

2. Central Statistics Office, Ireland. Irish Life Tables No.16, 2010-2012 [Internet]. Available from: https://pdf.cso.ie/www/pdf/20171121010152_Irish_Life_Tables_No_16_20102012_full.pdf

3. HSE Primary Care Reimbursement Service. Expenditure reports for the Dental Treatment Service Scheme. Dublin, Ireland;

4. Husereau D, Drummond M, Petrou S, Carswell C, Moher D, Greenberg D, et al. Consolidated health economic evaluation reporting standards (CHEERS)—explanation and elaboration: a report of the ISPOR health economic evaluation publication guidelines good reporting practices task force. Value Health. 2013;16(2):231–50.
